# Tadpole growth rates and gut bacterial community: Dominance of developmental stages over temperature variations

**DOI:** 10.1371/journal.pone.0292521

**Published:** 2023-10-05

**Authors:** Jun-Kyu Park, Woong-Bae Park, Yuno Do

**Affiliations:** Department of Biological Sciences, Kongju National University, Gongju, Republic of Korea; Bigelow Laboratory for Ocean Sciences, UNITED STATES

## Abstract

Tadpoles present an intriguing model system for studying the regulation and selection of gut microbiota. They offer a unique perspective to enhance our understanding of host-microbiota interactions, given their capacity to alter the dynamics of the gut microbial community by interacting with multiple environmental factors within a complex life cycle. In this study, we comprehensively investigated variations in growth rate and gut bacterial community in relation to temperature differences during the complex process of amphibian metamorphosis. Higher temperatures prompted tadpoles to metamorphose more rapidly than at lower temperatures, but the impact on size and weight was minimal. Differences in temperature were not associated with gut bacterial diversity, but they did affect certain aspects of beta diversity and bacterial composition. However, the developmental stage invoked greater heterogeneity than temperature in gut bacterial diversity, composition, and functional groups. These findings suggest that inherent biological systems exert stronger control over an organism’s homeostasis and variation than the external environment. Although results may vary based on the magnitude or type of environmental factors, metamorphosis in tadpoles greatly influences their biology, potentially dominating microbial interactions.

## Introduction

The gut microbiome has garnered substantial attention in scientific research due to its critical role in host health, including metabolic functions such as nutrient breakdown, vitamin production, and energy extraction [1, 2]. Beyond metabolic support, gut microbiomes also fine-tune immune responses, striking a balance between immune activation and tolerance [3]. These microbial communities are dynamic players in host physiology, contributing to disease resistance, digestion, and nutrient absorption [4].

Most gut microbiome studies have focused on endothermic vertebrates [5, 6]. Recently, this research domain has started to explore ectothermic creatures like insects, fish, amphibians, and reptiles [7–10]. Considering that roughly 99% of all animal species and about 75% of vertebrates are ectothermic [11], the necessity for greater gut microbiome research within this group is clear. In particular, comprehending microbiome-host interactions in ectothermic animals is challenging, as their body temperature changes with their environment and they display a wide array of lifestyles [12, 13].

Tadpoles offer a compelling model system to explore these complexities. Undergoing remarkable transformations during their metamorphosis, they transition from an herbivorous aquatic lifestyle to a carnivorous terrestrial one [14]. This change also manifests in their gut physiology, with notable alterations in stomach acidity, small intestine length, and hindgut expansion [15, 16]. These unique life stages offer a valuable lens through which to study gut microbiome dynamics [13], as well as their interactions with various environmental factors affecting the tadpole’s development.

The physical environment, particularly the element of temperature, has a profound effect on frog physiology, growth, and behavior of frogs [17, 18]. Metabolism, growth, and even development rates of frogs, specially, tadpoles is intimately intertwined with environmental temperatures [19, 20]. The impact of temperature is so significant that it can modulate the pace and success of amphibian metamorphosis. Recent studies suggest an extended influence of temperature that reaches beyond the host to its microbiome [21]. Temperature, in particular, is identified as a potential driver of changes in this intricate ecosystem’s structure and function. In aquatic habitats, oscillations in temperature can instigate significant shifts in both microbial diversity and metabolic activity [12, 20]. These changes carry with them the potential to redefine the existing paradigm of host-microbiome interactions.

The primary hypothesis of the current study is based on the idea that variations in temperature impact tadpole growth rates, which, in turn, influence the composition and functionality of their gut bacterial communities. This premise stems from the understanding that temperature sensitivities in tadpole growth could lead to corresponding shifts in the gut microbiome. As tadpoles grow and undergo metamorphosis, the physiological and dietary shifts are expected to be reflected in the structure of their gut bacterial community. The study aims to explore this relationship further, investigating how temperature-induced variations in tadpole growth rates lead to subsequent changes in their gut bacterial communities. The objective is to determine if temperature-driven changes in growth rates significantly affect these physiological transitions, thereby leading to alterations in the gut bacterial community.

## Material and methods

### Experimental setup

In June 2022, we sourced 15 mating pairs of Japanese tree frogs (*Dryophytes japonicus*) from Gongju-si, Chungcheongnam-do, resulting in the procurement of 15 egg clutches. The collected clutches were relocated and nurtured in five equally dimensioned containers (measuring 630mm × 430mm × 320mm), each accommodating three egg clutches. Our incubation environment, characterized by a temperature of 22±1°C, was equipped with an air generator and maintained under a 12 h light/12 h dark cycle facilitated by 3W LED lights.

Upon reaching Gosner stage 26 in their development, as defined by the Gosner stages [22], the tadpoles were divided into two distinct groups and exposed to different water temperatures, 24°C and 32°C respectively. Tadpoles were randomly selected, with five individuals taken from each container. A total of 25 tadpoles, drawn from five containers, were placed in a single cage measuring 480 mm × 300 mm × 290 mm. Each temperature condition (24°C and 32°C) had six replicate cages. Of these six cages, five were used directly in the experiment, while one cage was reserved as a replenishment set. This reserve cage was used to replace individuals that either died or were in poor condition.

An electric heater was used to maintain the water temperature, which was checked every 12 hours. To ensure accurate temperature control, the water thermometer inside each cage was alternated with the electric heater’s temperature gauge.

The tadpoles were fed fish feed (Tetra bits Complete, Tetra, Germany; Protein 47.5%, Fat 4.8%, Phosphorus 1.6%, Calcium 1.4%) once every three days. The feed amounted to 8% of their body weight per feeding session. Given that a diet comprising 21% protein at 8% of body weight is considered to be moderate food restriction [23, 24], we deemed this feeding regimen sufficient. Although the general guideline for this type of fish feed is to provide an amount the fish can consume within five minutes, we left any leftover feed in the cage until the next day to ensure adequate nourishment. Remaining food was removed the following day.

Water in the cages was replaced with sterilized deionized water once a week. All experimental procedures involving animals were carried out in accordance with regulations and were approved by the Experimental Animal Ethics Committee of Kongju National University (KNU_2022–01).

### Sampling procedures

**Fig 1:**
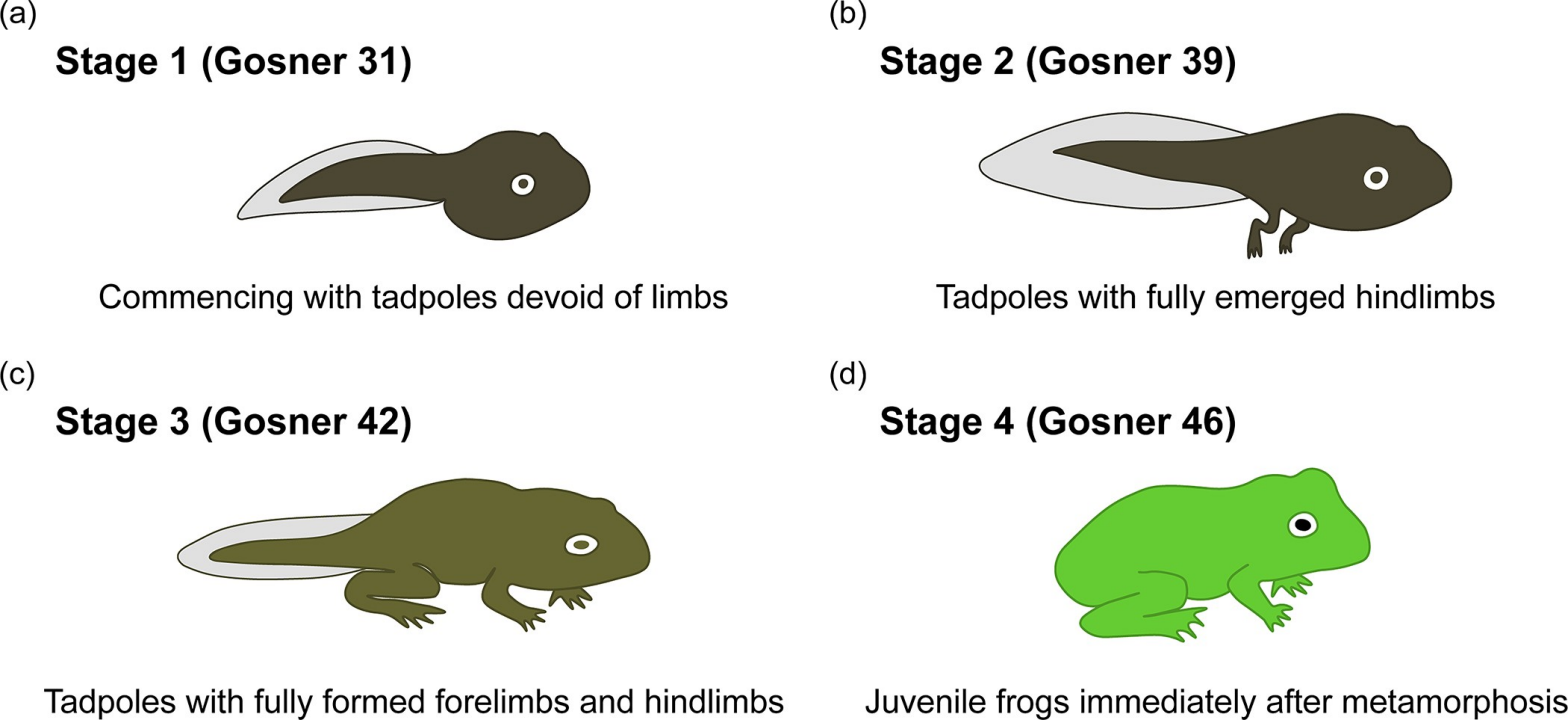
The four developmental stages of tadpoles sampled in this study. (a) Stage 1: Gosner stage 31 tadpole with no limbs; (b) Stage 2: Gosner stage 39 tadpole with fully developed hindlimbs; (c) Stage 3: Gosner stage 42 tadpole with both fully formed hindlimbs and forelimbs; (d) Stage 4: Juvenile frogs at Gosner stage 46 immediately after metamorphosis.

We implemented a four-stage sampling strategy, categorized according to Gosner stages [22]. The sampling commenced with limbless tadpoles at Gosner stage 31 (Stage 1), progressed to tadpoles with fully emerged hindlimbs at Gosner stage 39 (Stage 2), followed by tadpoles with fully developed forelimbs and hindlimbs at Gosner stage 42 (Stage 3), and concluded with juvenile frogs immediately post-metamorphosis at Gosner stage 46 (Stage 4). Each stage of sampling included three tadpoles per cage, yielding a total sample size of 15 individuals per group (Fig 1).

Tadpoles were humanely euthanized using a solution of 1 g/L MS-222 (tricaine methane sulfonate), balanced with sodium bicarbonate, for subsequent length and weight measurements, as well as gut dissection for bacterial community analysis. Post-euthanasia, each tadpole was blotted with sterile gauze, weighed, and photographed to capture length data. Utilizing ImageJ software [25], we recorded tadpole body lengths—from snout to tail tip—to an accuracy of 0.001 mm. Weights were determined using a digital balance accurate to 0.001 g.

After collecting length and weight data, the tadpoles were transferred to a sterile bench for dissection. The start of the small intestine and the end of the large intestine were meticulously removed using sterile scissors and tweezers. To prevent cross-contamination, each tadpole was dissected using a unique pair of autoclaved scissors and tweezers. The removed gut samples were subsequently moved to tubes containing beads included in the Qiagen DNeasy Powersoil Pro Kit (Qiagen, Hilden, Germany).

### DNA extraction and gene amplicon

We extracted DNA using the DNeasy PowerSoil Pro Kits from QIAGEN. Samples were combined with lysis buffer in a tube and then homogenized for 5 minutes at a speed of 30 m/s using the TissueLyser II (Qiagen, Hilden, Germany). We followed the kit’s instructions for DNA extraction. The quality of the extracted DNA was verified by electrophoresis and quantified with a DeNovix-QFX fluorometer (Denovix Inc., Wilmington, DE, USA) alongside a QFX dsDNA High Sensitivity Assay Kit (Denovix Inc., Wilmington, DE, USA). We ensured the genomic DNA from gut bacteria in all samples exhibited no abnormalities in quality and concentration.

The Polymerase chain reaction (PCR) was performed using primers targeting the 16S ribosomal RNA gene (16S rRNA gene) V4 region, which also incorporated the overhang adapter sequence provided by Illumina. The primers utilized were the forward primer (515F:5′—TCGTCGGCAGCGTCAGATGTGTATAAGAGACAGGTGCCAGCMGCCGCGGTAA—3′) and the reverse primer (806R:5′-GTCTCGTGGGCTCGGAGATGTGTATAAGAGACAGGGACTACHVGGGTWTCTAAT—3′). We executed PCR conditions as specified in "Preparing 16S Ribosomal RNA Gene Amplicons for the Illumina MiSeq System" [26] using the KAPA HiFi HotStart Ready Mix (Kapa Biosystems Inc., Wilmington, USA). The primary PCR product was purified using the Agencourt AMPure XP PCR purification system (Beckman Coulter, Brea, CA, USA). Subsequently, an index PCR was performed with the Nextera XT Index Kit (Illumina, San Diego, CA, USA). The index PCR products underwent a similar purification process. We assessed the final PCR product for size and quality through electrophoresis and determined its concentration with the same DeNovix-QFX fluorometer and assay kit as before. Samples were then diluted, pooled, and analyzed on the Illumina MiniSeq system (Illumina, Inc., San Diego, CA, USA). This extraction procedure focused on the 16S rRNA V4 region, a widely recognized marker for tracking bacterial and archaeal phylogenies. Upon extraction completion, the DNA samples were sequenced using the MiniSeq Illumina sequencing system.

### Data processing

The resulting sequencing data were processed using the QIIME2 pipeline (version 2022.2) [27]. Within this framework, we applied a filter focused on the Bacteria domain, effectively narrowing our analysis to bacterial communities in our samples. Quality filtering, barcode-based sample assignment, noise removal, merging of denoised paired-end sequences, utilization of both forward and reverse reads, and construction of feature tables were conducted using DADA2 plugins. We employed the QIIME feature table rarefy function to minimize differences in sequencing depths across samples, setting it at 95% of the minimum sequencing depth. Trimming parameters were configured based on Demux visualizations. Taxonomic assignments were performed using BLAST searches against the SILVA 123 database.

For subsequent data analysis and visualization, we used the microeco package for R (version 4.2.2) [28]. This allowed us to calculate various alpha diversity indices such as Observed, Shannon, InvSimpson, and Phylogenetic Diversity (PD), as well as to conduct beta diversity analysis. We analyzed differences in gut bacterial composition using permutational multivariate analysis of variance (PERMANOVA), setting the number of permutations at 999. The results were visualized through Principal Coordinates Analysis (PCoA) based on Bray-Curtis distance. We employed the Shapiro-Wilk normality test and Levene’s equal variance test to identify the appropriate analytical model for differences in body length, body weight, development rate, and alpha diversity indices of the gut bacterial community among eight groups (two different temperatures × four developmental stages). Consequently, we implemented a Kruskal-Wallis test followed by a post hoc Dunn test.

The relationships between various sample groups were visualized using Venn diagrams, focusing on shared and unique species. Further statistical analysis was carried out using Linear Discriminant Analysis (LDA). We set the threshold of the histogram of LDA scores to 3.0 to identify gene function taxa differences. The group from Stage 1 at 32°C served as the standard for assessing differences in relative abundance, as reflected by the Linear Discriminant Analysis Effect Size (LEfSe). This approach enabled us to identify functionally distinct bacterial communities that contribute significantly to diversity among the sample groups. We utilized the Tax4Fun R package [29] for functional prediction of the 16S rRNA datasets.

## Results

### Body length and weight across stages and temperatures

**Fig 2:**
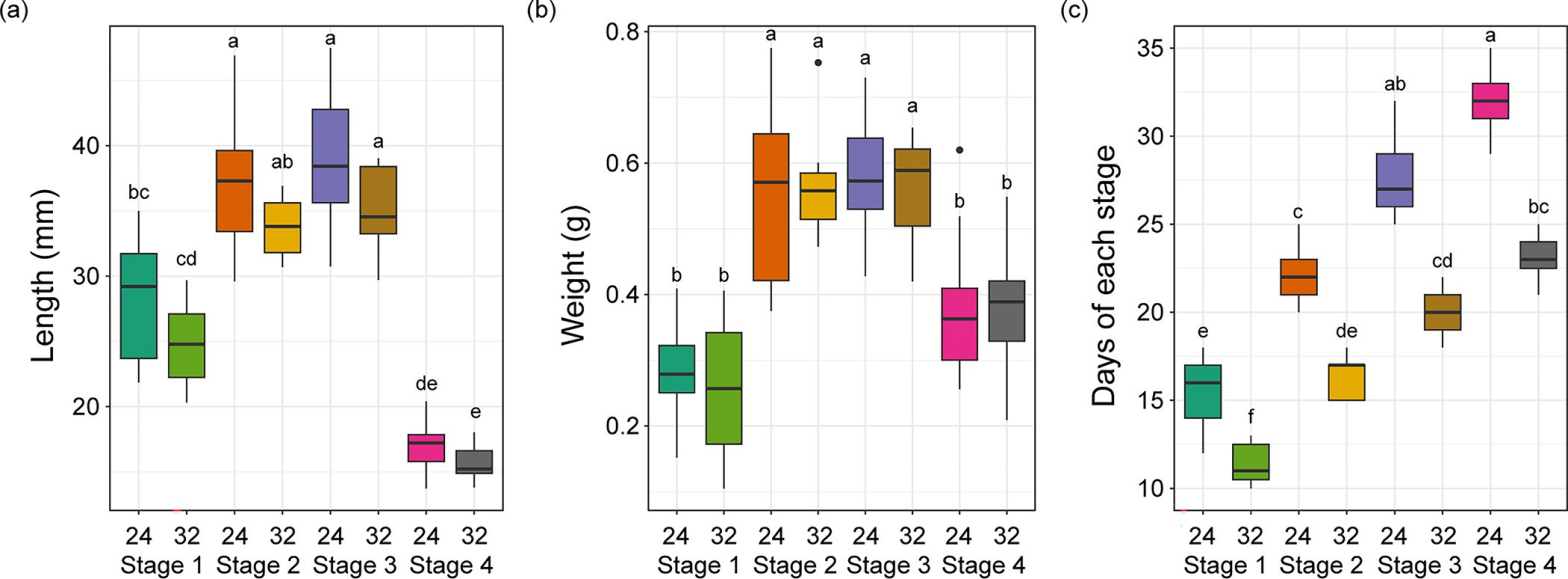
Box plot comparing body length, body weight, and days at each stage by temperature and developmental stage. The median value is indicated by the central line, the first quartile by the bottom box, the third quartile by the top box, the range within 1.5 interquartile by the top and bottom lines, and outliers by the circles. Significant differences (*p* < 0.05) were determined using Dunn’s post hoc test following Kruskal-Wallis test, and are indicated by difference of lowercase letters.

Significant differences were observed in tadpole body length and weight across various developmental stages, regardless of temperature conditions (Kruskal-Wallis test, Length: H_(df = 7)_ = 98.951, *p* < 0.001; Weight: H_(df = 7)_ = 81.901, *p* < 0.001). However, within each developmental stage, there were no significant differences in body length and weight between the 24°C and 32°C conditions (Dunn’s post hoc, *p* > 0.05). An increase in both body length and weight was evident from Stage 1 to Stage 2 (Dunn’s post hoc, *p* < 0.05). Stages 2 and 3 showed no significant difference in these metrics (Dunn’s post hoc, *p* > 0.05). In contrast, Stage 4 exhibited a decline in both body length and weight (Dunn’s post hoc, *p* < 0.05), suggesting that metamorphosis might lead to a reduction in overall body size and mass (Fig 2A and 2B).

Developmental progress, assessed in terms of days post-hatching, varied significantly across the different stages (Kruskal-Wallis test, H_(df = 7)_ = 112.680, *p* < 0.001). Tadpoles kept at 32°C developed more quickly through all stages compared to those at 24°C (Dunn’s post hoc, *p* < 0.05) (Fig 2c). This observation suggests that a warmer environment may accelerate the developmental progression of tadpoles.

### Alpha and beta diversity

**Fig 3:**
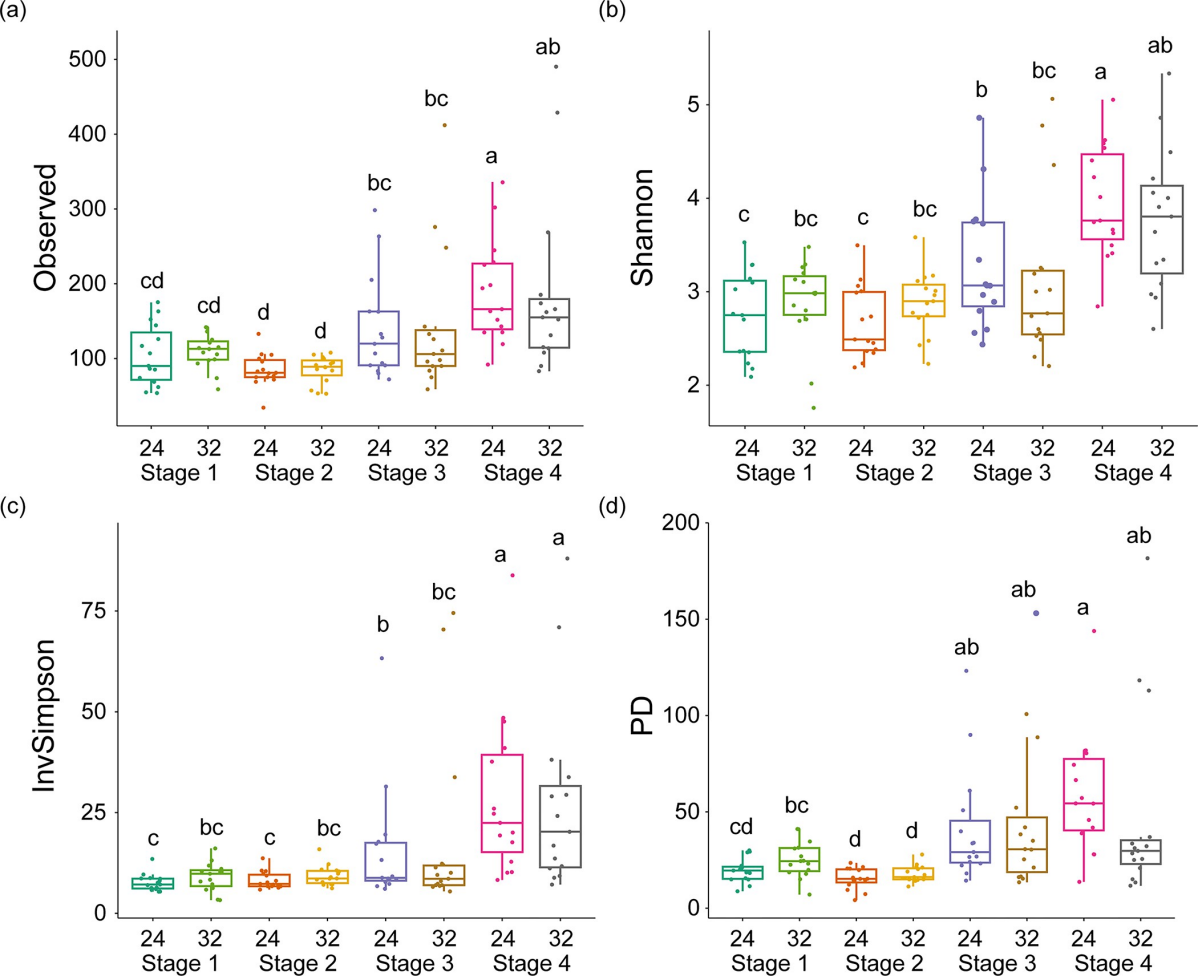
Alpha diversity of the gut bacterial community in frogs by temperature and developmental stage. (a) Observed species; (b) Shannon index; (c) Inverse Simpson index (InvSimpson); (d) Phylogenetic diversity whole tree (PD). Box plots represent the median value (central line), first and third quartiles (bottom and top of boxes), and range within 1.5 interquartile (bottom and top of lines). Significant differences (*p* < 0.05) were determined using Dunn’s post hoc test following Kruskal-Wallis test, and are indicated by difference of lowercase letters.

Upon examining the tadpoles kept at 24°C, we noted statistically significant variations in Observed (Kruskal-Wallis test, H_(df = 7)_ = 44.332, *p* < 0.001), Shannon (Kruskal-Wallis test, H_(df = 7)_ = 43.395, *p* < 0.001), InvSimpson (Kruskal-Wallis test, H_(df = 7)_ = 43.102, *p* < 0.001), and PD indices (Kruskal-Wallis test, H_(df = 7)_ = 48.252, *p* < 0.001) across the developmental stages (Fig 3A–3D). Within each stage, the diversity indices showed no significant differences related to temperature (Dunn’s post hoc, *p* > 0.05). Stage-specific differences were the primary contributors to variations in gut bacterial diversity indices (Dunn’s post hoc, *p* < 0.05). Generally, diversity indices increased in Stages 3 and 4 compared to Stages 1 and 2. Stage 4 exhibited the highest diversity in all indices except for PD, while Stage 2 had the lowest diversity in Observed and PD indices (Dunn’s post hoc, *p* < 0.05).

Diversity variations across developmental stages were more pronounced at 24°C than at 32°C. At 24°C, bacterial diversity between Stages 1 and 2 showed no significant difference (Dunn’s post hoc, *p* > 0.05), while a significant increase was observed from Stage 2 to both Stages 3 and 4 (Dunn’s post hoc, *p* < 0.05). In contrast, at 32°C, gut bacterial diversity indices remained relatively stable from Stage 1 to Stage 3 (Dunn’s post hoc, *p* > 0.05), with only a slight increase seen in Stage 4. For PD, the levels of bacterial diversity were consistent in Stages 1, 3, and 4, except for a reduction in Stage 2 (Dunn’s post hoc, *p* < 0.05).

**Fig 4:**
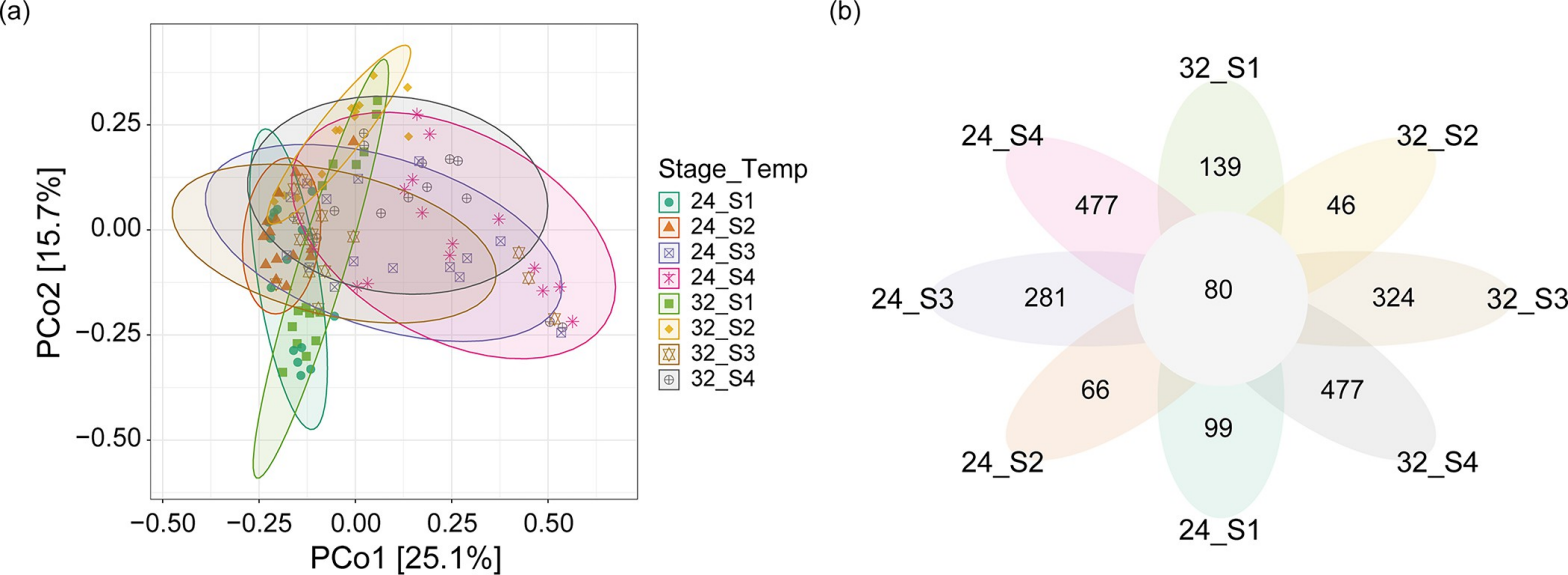
Beta diversity of the gut bacterial community in frogs by temperature and developmental stage. (a) Scatter plot of principal coordinate analysis (PCoA) based on Bray–Curtis dissimilarity; (b) Venn diagram showing the overlap of gut bacterial operational taxonomic units (OTUs).

Permutational multivariate analysis of variance (PERMANOVA) delineated unique clusters according to both temperature (R^2^ = 0.033, F_(1)_ = 4.874, *p* < 0.001) and developmental stage (R^2^ = 0.198, F_(3)_ = 9.885, *p* < 0.001) of the tadpoles, as visualized in Fig 4A through principal coordinates analysis (PCoA). The first axis (PCo1), responsible for 25.1% of the total variation, effectively segregated the samples based on developmental stage. In this context, the clusters corresponding to Stages 1 and 2 were markedly different from those associated with Stages 3 and 4, irrespective of the temperature conditions. Meanwhile, the second axis (PCo2), accounting for 15.7% of the observed variation, mainly separated the samples by temperature, but this was only evident in Stage 2.

The Venn diagram in Fig 4B elucidated the unique and shared operational taxonomic units (OTUs) among the tadpoles at different temperatures and developmental stages. In the 32°C group, tadpoles at Stage 4 exhibited the highest count of unique OTUs (477), followed by those at Stage 3 with 324 unique OTUs. Stage 2 had the fewest, with only 46 unique OTUs. In the 24°C group, Stage 4 once again led with 477 unique OTUs, followed by Stage 3 with 281. Stages 1 and 2 lagged behind, displaying considerably fewer unique OTUs. Across all conditions, a total of 80 OTUs were universally present.

### Composition of gut microbiome of tadpole

**Fig 5:**
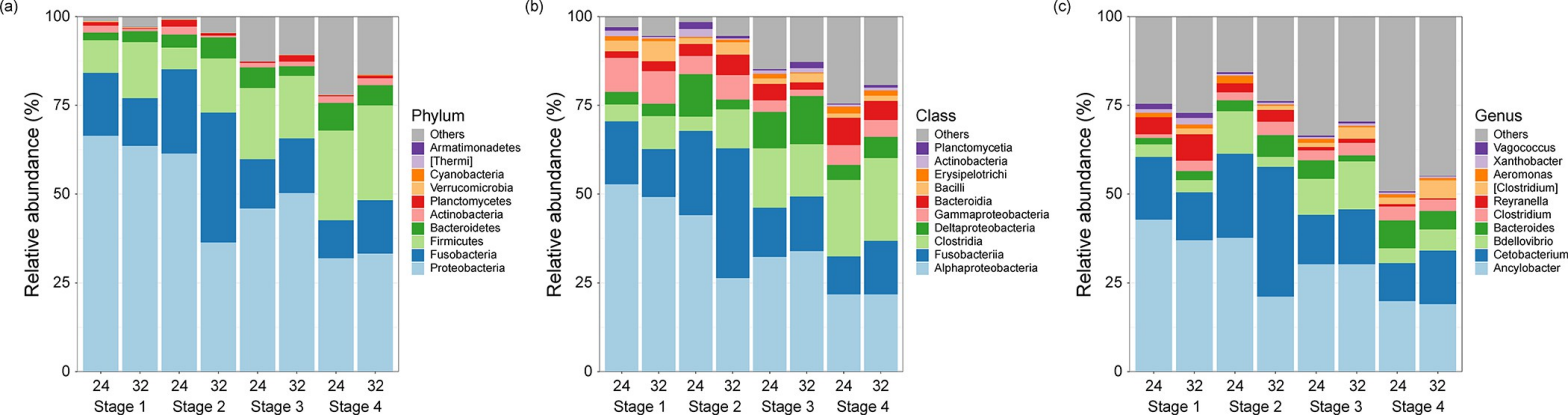
Relative abundance of the gut bacterial community in tadpoles by temperature and developmental stage: (a) Phylum level of the gut bacterial community; (b) Class level of the gut bacterial community; (c) Genus level of the gut bacterial community.

At 24°C, the gut bacterial community was predominantly populated by Proteobacteria, though their representation significantly reduced as the developmental stages advanced. The proportions of Firmicutes, Fusobacteria, and Bacteroidetes notably increased during the stages. In contrast, at the higher temperature of 32°C, proteobacteria remained dominant but with a slightly reduced ratio. Fusobacteria showed a significant increase at stage 2, and a consistent presence of Firmicutes was observed across stages (Fig 5A).

At 24°C, the gut bacterial community was heavily dominated by Alphaproteobacteria, yet this dominance lessened as the tadpoles progressed through their developmental stages. In contrast, the representation of Bacilli, Bacteroidia, Clostridia, and Deltaproteobacteria increased significantly as the stages advanced. When the temperature was raised to 32°C, Alphaproteobacteria remained the most dominant class, although their presence slightly decreased over the stages. The proportions of Bacilli and Bacteroidia remained relatively stable across all stages, while Clostridia and Deltaproteobacteria showed an increase (Fig 5B).

In the developmental stages of tadpoles at 24°C, Ancylobacter was the most dominant genus. Its prevalence declined over time, while genera such as Cetobacterium, Bdellovibrio, and Bacteroides saw a modest upsurge. Particularly, Clostridium showed a steady increase across the stages. In contrast, at 32°C, Ancylobacter maintained its dominant position, despite a slight reduction through the stages. Cetobacterium peaked at the second stage before tapering off. The prevalence of Bdellovibrio increased, particularly at the third stage, whereas Bacteroides exhibited a fluctuating pattern. Just as in the cooler condition, Clostridium displayed a continual rise. Irrespective of the temperature, the genus Reyranella exhibited a significant decline across the stages (Fig 5C).

### Functional profile of gut bacterial communities

**Fig 6:**
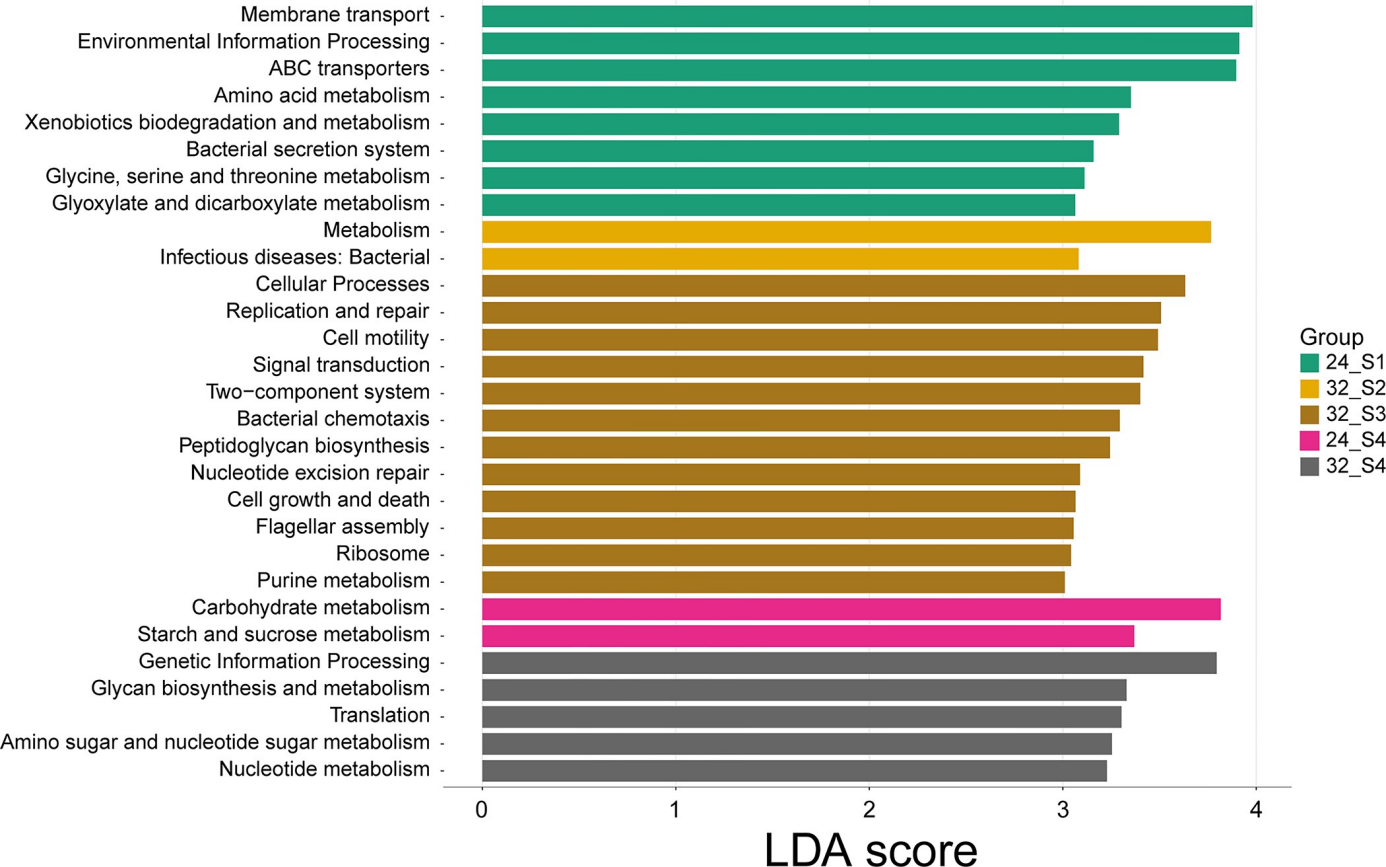
Linear discriminant analysis (LDA) scores reflecting the differences in relative abundance of functional groups within the gut bacterial community of tadpoles, by temperature and developmental stage.

At 24°C, the gut bacterial communities of Stage 1 tadpoles exhibited significant functionality in membrane transport, environmental information processing, ABC transporters, and amino acid metabolism, notably glycine, serine, and threonine metabolism (Fig 6). Additionally, these communities were engaged in xenobiotics biodegradation and metabolism, bacterial secretion systems, and glyoxylate and dicarboxylate metabolism. Moving to stage 4 at the same temperature, the gut microbiota displayed predominant functionality in carbohydrate metabolism, particularly starch and sucrose metabolism.

Contrastingly, at 32°C, the gut bacterial communities demonstrated different dominant functions (Fig 6). Stage 2 tadpoles had communities primarily functioning in general metabolism and bacterial infectious diseases. However, by stage 3, these communities had diversified, showing prominent involvement in various cellular processes including replication and repair, cell motility, signal transduction, and other related functions. By stage 4, the bacterial communities were largely associated with genetic information processing, glycan biosynthesis and metabolism, translation, amino sugar and nucleotide sugar metabolism, and nucleotide metabolism.

## Discussion

**Fig 7:**
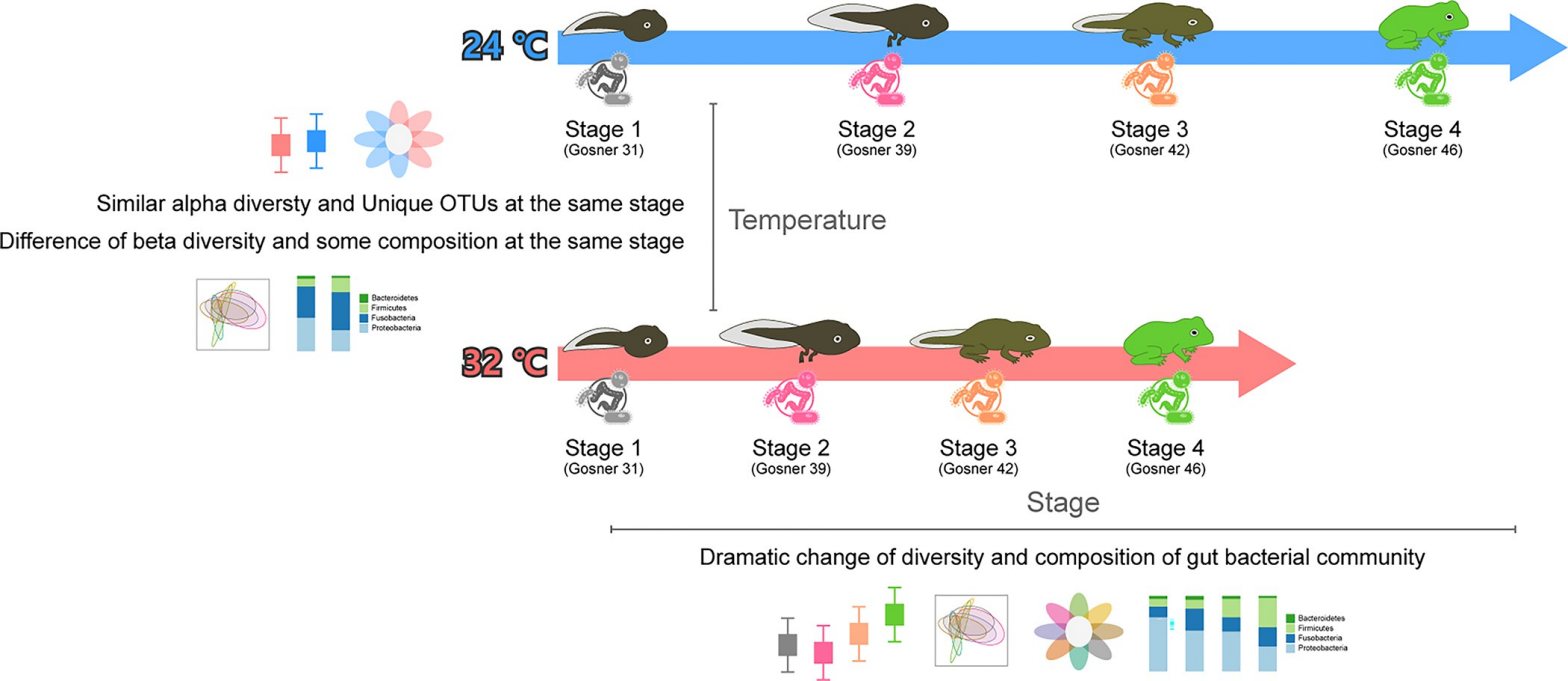
Summary of the experiment’s results. Despite some variations in the gut bacterial community due to temperature differences, the developmental stage was the most significant factor influencing changes in the gut bacterial community of tadpoles.

This study provided a comprehensive examination of the influence of temperature on tadpole growth and the ensuing impact on their gut bacterial communities across various developmental stages (Fig 7). The observations reaffirmed the dynamic interplay between environmental factors, physiological adaptations, and the gut microbiome composition, illuminating their collective roles in the complex process of amphibian metamorphosis.

The results deviated from our initial expectations. While temperature fluctuations did alter the pace of tadpole development, they didn’t impact the overall body size or weight. This suggests that for amphibian species spanning vast geographical areas or those breeding over elongated periods, temperature-driven responses might differ, even within the same species. Such variations are likely influenced by factors such as specific habitat conditions and the onset of breeding seasons [30–33]. This leads us to speculate that intrinsic physiological elements, including nutrient intake, growth efficacy, and energy assimilation, may play a more pivotal role in governing growth and developmental trajectories than merely external environmental cues.

Taking the Japanese tree frog as an example—a species prevalent across East Asia and predominantly breeding between spring and summer [34]—our observations might not be applicable to its populations experiencing different climatic conditions or breeding timelines. In essence, monitoring gut microbiota shifts in connection to temperature gradients and geographical spread can shed light on the adaptive strategies amphibians deploy against climatic changes. This becomes even more critical given the fact that climate change has been a dominant factor in the dwindling amphibian populations [35]. For species restricted by specific temperature margins or confined habitats, like those in high-altitude regions [36, 37], understanding these adaptive responses becomes paramount. In-depth research into immune responses, physiological endurance, and gut microbiota evolution across species spectrum, ranging from the susceptible to the resilient, will enrich our knowledge about adaptive mechanisms. This will further refine our perspective on how different species might confront the challenges posed by climate change [13].

While our study found no significant changes in gut bacterial diversity due to temperature, it did observe shifts in the composition of the gut bacterial community. These findings align with previous research which reported temperature-induced changes in the gut microbiome, albeit at specific developmental stages (Gosner stages 38–39). In similar conditions, tadpoles raised at cooler (18°C) and warmer (28°C) temperatures—analyzed at the same Gosner stages 38–39—showed no differences in microbial diversity but did exhibit variations in community composition. Moreover, at these same stages, Proteobacteria were more abundant in tadpoles at cooler temperatures [38]. Our study corroborated these observations, specifically at Gosner stage 39. However, the temperature sensitivity was most pronounced at this stage, diminished at stage 31, and reversed entirely at stages 42 and 46, suggesting that temperature effects are stage-dependent during tadpole metamorphosis.

Additionally, previous literature suggests that higher temperatures tend to increase the heterogeneity of microbial communities among individual hosts [39, 40]. In our study, this heterogeneity was more markedly influenced by changes in developmental stage than by temperature variations. Particularly at Gosner stages 42 and 46, we observed a significant increase in the variation of alpha diversity within the gut bacterial communities. This observation is supported by both the Principal Coordinates Analysis (PCoA) and the Venn diagram depicting unique Operational Taxonomic Units (OTUs). Consequently, it appears that changes in the composition and diversity of gut bacterial communities are more sensitive to developmental transitions than to temperature fluctuations.

Throughout all stages, Proteobacteria dominated the gut microbiota of tadpoles. However, as tadpoles developed, there was a decline in Alphaproteobacteria, a subset of Proteobacteria. This decline was counterbalanced by a rise in classes such as Bacilli, Bacteroidia, Clostridia, and Deltaproteobacteria. Such bacterial shifts likely correspond to the significant physiological and dietary adaptations that tadpoles undergo during metamorphosis [13]. The alterations in the bacterial community composition might be aiding the tadpoles throughout their transformation into adult frogs [7, 41]. Most notably, the major shifts in bacterial diversity coincided with the stages that featured significant changes in the anatomy of the tadpole gut, suggesting a collaborative relationship between gut structure and its microbiota [13, 41]. As tadpoles metamorphosed, their bacterial community evolved, potentially to support their evolving physiological and dietary demands. This highlights the dual influence of both the external environment and the host’s physiological state on the microbiome.

Concurrently, the gut bacterial communities’ functional roles showed variations across the different developmental stages and temperature conditions. The functional attributes of the gut microbiome evolved to meet the host’s metabolic needs specific to each stage [41]. During the initial stages, the prominent functions were related to membrane transport, environmental information processing, and amino acid metabolism. However, as the tadpoles neared maturity, there was an evident shift towards carbohydrate metabolism, indicating a connection between the microbiome’s functional roles and the tadpoles’ shifting nutritional requirements [41].

Of note is the observation that 80 OTUs of gut bacterial groups were shared across all developmental stages. This count is approximately double the unique OTUs identified in tadpoles at Gosner stage 39. While the number of unique OTUs surged by Gosner stages 42 and 46, the 80 shared bacterial groups remained stable. This pattern suggests that the gut microbiota is not entirely reset during tadpole metamorphosis. Our findings indicate that while tadpoles continuously integrate new microbes and experience shifts in composition during development and metamorphosis, they also retain essential existing microbes. Future in-depth studies focusing on the roles these consistent bacterial groups play during metamorphosis will further elucidate the intricacies of host-microbe dynamics during tadpole growth.

## Conclusion

The influence of developmental stage on tadpoles’ physical conditions, as well as their gut bacterial diversity and composition, appears to outweigh the effects of temperature variations. Nevertheless, populations of the same species collected from different latitudes or breeding seasons could show distinct sensitivities, highlighting the need for additional research. Our data suggest that biological growth mechanisms, responsive to diverse environments, serve to maintain a level of consistency even when external conditions vary. This observation is further corroborated by the variability in gut bacteria across developmental stages. Although temperature is a significant environmental variable for ectothermic vertebrates, its impact seems particularly pronounced in tadpoles. This is likely because tadpole development and metamorphosis bring about substantial changes in various biological facets, including behavior, physiology, and morphology. Despite the potential for variation depending on the nature or intensity of external environmental factors, internal biological control systems may often exert a more potent influence on an organism’s homeostasis and diversity than their external conditions.
